# An intragenic distribution bias of DNA uptake sequences in *Pasteurellaceae *and *Neisseriae*

**DOI:** 10.1186/1745-6150-3-12

**Published:** 2008-03-27

**Authors:** Mark WJ van Passel

**Affiliations:** 1 Laboratory of Microbiology, Wageningen University, Dreijenplein 10, 6703 HB, Wageningen, The Netherlands

## Abstract

Most sequenced strains from *Pasteurellaceae *and *Neisseriae *contain hundreds to thousands of uptake sequence (US) motifs in their genome, which are associated with natural competence for DNA uptake. The mechanism of their recognition is still unclear, and I searched for intragenic location patterns of these motifs for clues about their distribution. In all cases, one orientation of the US has a higher occurrence in the reading frame, and in all *Pasteurellaceae*, the US and the reverse complement motifs are biased towards the gene termini. These findings could help design experimental set-ups to study preferential DNA uptake, thereby further unravelling the phenomenon of natural competence.

This article was reviewed by Arcady Mushegian and I. King Jordan.

## Findings

Gene repertoires are not stable features of prokaryotes [1], and it has been suggested that most genes have been affected by horizontal gene transfer at some point during their evolutionary history [2]. Environmental DNA not only serves as a potential nutrient for bacteria [3], but also represents an important substrate for the acquisition of new coding sequences via incorporation into the genome [4]. The ability to take up DNA and thereby acquiring new genes from the environment is termed natural competence, but this trait is not shared between all bacteria to the same extent. This implies that for some bacteria, or in selected environments, natural competence is a more favourable ability.

In the *Pasteurellaceae *and *Neisseriaea*, natural competence has been linked to specific DNA motifs that are substantially overrepresented in the genome. The DNA Uptake Sequence (DUS) 5'-ATGCCGTCTGAA-3' in *Neisseria *species occurs almost 1,500 times in the genome [5,6], and represents approximately 2% of the genome. The Uptake Signal Sequence (USS) in most *Pasteurellaceae *is 5'-AAAGTGCGGT-3' [7,8], although a different motif is encountered in a subclade of this family [8]. If the conspecific DNA uptake sequence (or US, as I refer to them collectively) is present in the donor DNA, it increases the transformation efficiency many fold [9]. The exact mechanism how these motifs enhance DNA uptake remains unclear. It has been postulated that USs may be involved in some sort of transformation-barrier, as they selectively increase DNA uptake from a source containing the same US [10], even though this US can be shared by distantly related *Pasteurellaceae *species [11]. Alternatively, these genomically overrepresented motifs were suggested to be associated with the replication and repair mechanisms due to their genome distribution [12], but no general orientation bias has ever been observed for these sequences in the genome sequences in which they reside. Another study proposed a role for transcription termination, due to the occurrence of inverted repeat forms of the intergenic USs, allowing the formation of hairpins [6]. However, as a substantial fraction of the USs are not in this inverted repeat configuration, and because many USs are intragenic, the transcription terminator explanation is still contentious [13].

Despite research into the genomic distribution of these sequence motifs [6,14], no analyses have focused on the distribution patterns of USs within the protein coding regions. Recently, we identified an intragenic positional preference of homopolymeric tracts in prokaryotes, which suggested that there has been intensified selection against these mutagenic repeats in the middle of genes [15]. In the current study I test whether USs also show a skewed distribution in the protein coding genes of the *Pasteurellaceae *and *Neisseriae*.

**Figure 1:**
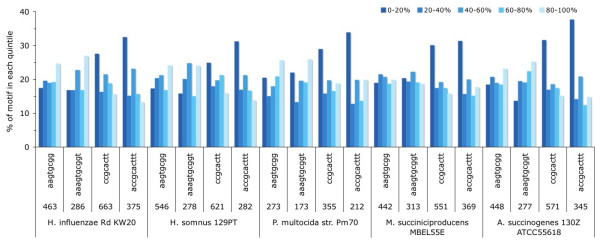
**The positional bias of the tested sequence motifs within genes.** In each genome, the genes were divided proportionally into five quintiles (with 0–20% denoting the initial one-fifth of the gene beginning at its 5' end, and 80–100% denoting the 3' segment), and the numbers of the sequence motifs were summed for each segment. The sequence motifs are depicted with different length stringencies (8 and 10 bp): the US is 5'-(A)AAGTGCGG(T)-3' (the left two lanes per genome sequence), whereas the rcUS is 5'-(A)CCGCACTT(T)-3' (the right two lanes per genome). The accession numbers of the five tested genome sequences are NC_000907, NC_008309, NC_002663, NC_006300 and NC_009655, respectively.

In the genes of the fully sequenced genomes from *Haemophilus influenzae*, *Pasteurella multocida*, *Mannheimia succiniciproducens *and *Actinobacillus succinogenes*, the reverse complement of the US is observed consistently more frequent than the US itself (for all tested genomes except *Haemophilus somnus*, p < 0.05 (Additional File 1), Fig. 1). However, the US and its reverse complement, the rcUS, show different distributions with respect to their location bias within genes. While the US is located predominantly at the 3'-end of open reading frames (except in *M. succiniciproducens*), the rcUS is located predominantly in the 5'-end of genes. In order to discern a trend in the distribution of the USs, I increased the stringency of the motif from 8 to 10 base pairs, and a pronounced increase of the location bias is observed for all genomes with respect to the rcUS, and for most genomes with respect to the US (Fig. 1). As expected, the sequenced genomes other *H. influenzae *strains show similar intragenic distribution biases of the US and rcUS (Additional File 1). The alternative US motif in *Actinobacillus pleuropneumoniae*, 5'-ACAAGCGGTC-3' [8], shows a different pattern. The most prevalent motif in coding sequences is the rcUS, which is slightly biased towards the 3' end of genes, whereas the US seems biased towards the 5' end of coding sequences (Additional File 2).

The US in *Neisseria *is unrelated to the motifs found in the *Pasteurellaceae*, and due to its specific sequence, it hinders straightforward distribution pattern analyses. Initially, I found a strong bias towards the 5' end of protein coding genes, until I detected that dozens of annotated open reading frames in neisserial genomes actually start with the US. In contrary to the US in the *Pasteurellaceae*, the neisserial US motif starts with the potential start codon ATG, which could result in incorrect annotations. Eliminating the 30 genes in *N. meningitidis *MC58 that start with an US shows a different pattern altogether; even though many more rcUS are present in protein coding genes than US (245 vs. 123, respectively, p < 0.05), there was no similarity with the distribution bias of the USs from the *Pasteurellaceae *(Additional File 3). However, the USs and rcUSs motifs in *Neisseriae *show different, though not significant (p > 0.05), distributions, with the USs apparently underrepresented in the 5' end of genes.

In the first ever genome-wide sequence motif analysis, Smith and co-workers found that the *H. influenzae *US was distributed apparently randomly over the genome [14], similar to later observations in the genome of *A. succinogenes *[16]. However, a somewhat smaller than expected number of these USs was found within the coding fraction of the *H. influenzae *genome [14]. This observation was recently complemented by Davidsen and co-workers who found a biased distribution of USs towards genome maintenance genes, such as replication, recombination and DNA repair, in different representative species from the phylogenetically distinct *Pasteurellaceae *and *Neisseria *[17]. They proposed that the observed bias of USs towards this class of genes was somehow connected to their function in these unrelated taxonomic groups, indicating convergent evolution. In this study I show that also within coding regions, the distribution of distinct competence-associated sequences is non-random. The increased incidence of these motifs in gene termini of the tested *Pasteurellaceae *species suggests that their presence at these locations could convey a selective benefit, as these distributions are thought to have arisen independently. The exact nature of this proposed selective benefit is unknown, but these observations could give rise to an experimental setup to examine potential differences in transformation efficiency, as genes flanked by USs in different orientations could be acquired with different efficiencies. However, experiments in *Actinobacillus actinomycetemcomitans *do not point in this direction [16].

Alternatively, the observed location biases may be caused by potential misannotations of transcriptional start sites (TSS). As TSS are still mostly assigned by bioinformatic procedures [18], inadequate annotations could be responsible for a skew in the distribution patterns. However, I do not expect this to negate the biased occurrence of US motifs in protein coding genes, as the observed bias is not restricted to the 5' end of genes.

## Competing interests

The author(s) declare that they have no competing interests.

## Authors' contributions

MWJvP conceived the experiment, performed the analyses, interpreted the results, wrote and revised the manuscript.

## Reviewers' comments

Referee 1: Arcady Mushegian, Stowers Institute, Kansas City, USA.

AM1: The GC-content of both US motifs is exactly 50%, which is close to the average GC-content of a *Neisseria *genome (51–52%?), but is not quite close to those of *Pasteurellaceae *(for example, 35–38% in *Haemophilus*).

But more relevant are the GC-contents values of each of the 5 quintiles of the ORF lengths (i.e., what is plotted in Fig. 1). Can all or some of the biases in the US distribution be explained by the bias of the underlying nucleotide frequency distribution?

*MWJvP1*: *I tested this for the Haemophilus influenzae Rd and Pasteurella multocida genome sequences. Considering all protein coding genes (>100 bp) in H. influenzae, I find that, on average, the GC% for the 5 quintiles are 37.8%, 39.0%, 38.9%, 38.8% and 37.3%, respectively. As the US has a GC% of 50%, one would assume to actually find lower numbers of these motifs in the first and last quintile of genes, based solely on the GC%. Instead, I find higher numbers. For P. multocida, a similar GC% progression is observed (39.5%, 41.1%, 41.1%, 41.3% and 39.8%, for each of the five quintiles, respectively)*.

AM2: All biases and overrepresentations should be accompanied by the appropriate statistics.

*MWJvP2*: *My apologies for not including these in the first draft. I apply a standard Chi-square goodness of fit test (4 degrees of freedom), with a null-hypothesis that these USs are represented proportionally (i.e., equally) in each quintile. I find for H. influenzae Rd a p-value < 0.05 that this distribution represents an equally proportionate distribution, so this null-hypothesis is rejected. I expand this chi-square for all sampled genomes *(Additional File 1) *and find that for the rcUS in every tested Pasteurellaceae genome the observed distribution is significantly different from the expected distribution (H0 = equally proportioned). As for the US, 5 out of 8 tested genomes show no significant distribution bias (p > 0.05), although similar patterns are observed with respect to their location preference*.

*I conclude that the tested rcUS motifs are not proportionally distributed in the gene quintiles. Only the full 10 bp motif was tested, and not the shorter (incomplete) sequence motifs, which show less of a bias, and were merely used in *Figure 1*to illustrate that the observed bias is not caused by a subset of the US sequence*.

*As for the counts of the US and the rcUS, I find for all genomes except H. somnus that they occur in significantly non-equal amounts in the protein coding regions of the tested genomes*.

*For Neisseria, a significantly different number of USs and rcUSs is observed in open reading frames (p < 0.05), but there is no significant difference (p > 0.05) between the intragenic location biases of the 12 bp US and the rcUS, although there is for the 10 bp US (p < 0.05)*.

Referee 2: I. King Jordan, Georgia Tech University, School of Biology, Atlanta, USA.

MWJ van Passel reports on biases in the distributions of uptake sequences (US) that mediate natural competence in Pasteurellaceae and Neisseriae. Within genes, the reverse complement of the US (rcUS) is found more frequently than the US, and the rcUS is biased towards the 5' ends of genes while the US is biased towards the 3' ends of genes. This is a noteworthy discovery and I support publication of the report as a Discovery Note in Biology Direct. I have several questions related mainly to the clarity of presentation.

IKJ1: The most important point is that the biases in the distributions need to be supported by some statistical analyses. Some sort of goodness-of-fit such as chi-square with an appropriate correction for multiple tests should suffice.

*MWJvP1*: *As reviewer 1 also asked for this addition, I would like to refer to the answer given above (MWJvP2 to AM2). I hope this adequately addresses this point*.

IKJ2: According to the author, intergenic US distributions have been evaluated previously, but this is the first report on the occurrences of intragenic US. To underscore the importance of this difference, it would help the reader to known what the relative frequencies of intergenic versus intragenic US for the species analyzed are.

*MWJvP2*: *It was Hamilton O. Smith et al. in 1995, who noted that only 65% of all USs occur in the ORFs, whereas ~86% of the H. influenzae Rd genome had been annotated as coding sequences. But Redfield et al. (2006) already counted fewer of these US (1115 instead of 1465). Therefore, we checked for the five genomes depicted in *Figure 1*the total counts of the (rc)US in the CDS and in the entire genome. For the genomes of H. influenzae Rd, H. somnus, P. multocida, M. succiniciproducens and A. sucinogenes, the percentages of (rc)USs in the coding regions are 59%, 70%, 55%, 55% and 47%, respectively, whereas their coding fractions represent 84%, 88%, 88%, 89% and 87%, respectively *(Additional File 4*depict these data*). *This suggests a non-random distribution of DNA uptake sequence motifs with respect to the coding fractions, similar as to what Hamilton O. Smith et al. found*.

IKJ3: In the discussion, the distribution of US across different functional classes of H. influenzae genes is mentioned. This is a very interesting point and a similar analysis for Pasteurellaceae and Neisseriae would strengthen this manuscript. Along these same lines, I was wondering if US sequences are over-represented among genes that have been horizontally transferred and/or are strain-specific.

*MWJvP3*: *This distribution of (rc)USs has been analyzed thoroughly by Davidsen et al. (2004), which was in fact performed for both Pasteurellaceae and Neisseriae (I hope to have clarified this now in the text). They concluded that for both Pasteurellaceae and Neisseriae, the motifs are more common in the functional group of genome maintenance genes (GMGs). I quote "These results imply that the high frequency of DUS in genome maintenance genes is conserved among phylogenetically divergent species and thus are of significant biological importance" (Davidsen et al., 2004). There is however some debate whether these genes are frequently transferred: no aberrant nucleotide composition has been detected, but then again, it has been suggested that these US promote conspecific DNA uptake. Therefore, I did not carry out an analysis for US occurrence in horizontally transferred genes in this project*.

### Minor points

IKJ4: In Figure 1, it would helpful to indicate which are the US and which are the rcUS.

*MWJvP4*: *A reference to which lanes are depict the USs and which depict the rcUSs is now incorporated in the legend of *Figure 1.

IKJ5: The data on increasing stringency of the motif from 8 to 10 bp is reported as being shown in Fig 1. Should this be Supplementary Figure S1 (*now *Additional File 2, *MWJvP*)?

*MWJvP5*: *I am sorry that this wasn't mentioned properly in the legend of *Figure 1. *In this figure, four lanes per genome are visible: the two on the left are the US with increasing stringency (the motif is depicted below the lane, 8 bp and 10 bp), and the two on the right are the rcUS with increasing stringency. I hope the legend is now clearer*.

IKJ6: The intragenic distributions of US and rcUS in H. influenzae are reported as 'data not shown'. Given that Biology Direct is an online journal, it is preferable to report these distributions as Supplementary Information.

*MWJvP6*: *As there are several different strains of H. influenzae sequenced, I thought it better not to include these strains next to the H. influenzae rd KW20, as they do not represent independent measurements. However, I did expect a reviewer to ask whether these patterns are also found in the other strains, which they are (although not as pervasive with respect to the US distribution). I have included *Additional File 1*with the counts of the motifs in all the Pasteurellaceae genomes I tested, so readers also have access to the individual counts per genome*.

## Supplementary Material

Additional file 1 Total counts and fractions of the US in the genes of 7 Pasteurellaceae genomes. Total counts and percentages of the different motifs (the US 5'-AAAGTGCGGT-3' and rcUS 5'-ACCGCACTTT-3') in the coding regions of 7 Pasteurellaceae genomes (the accession numbers are given for each strain). Click here for file

Additional file 2 The intragenic distribution of the *Actinobacillus pleuropneumoniae *uptake sequence.
The intragenic distribution of the alternative US (left three sub-graphs, increasing the motif stringency from 8 to 10 bases) and its reverse complement (next three sub-graphs, increasing the motif stringency from 8 to 10 bases) in the protein coding regions of *Actinobacillus pleuropneumoniae *(accession number NC_009053). The counts of the motifs are depicted underneath. Click here for file

Additional file 3 The intragenic distribution of the *Neisseria meningitidis *uptake sequence. The intragenic distribution of the US and its reverse complement (rcUS) in the protein coding regions of *Neisseria meningitidis *MC58 (accession number NC_003112). Genes that start with the US are excluded. The sequence and the counts of the motifs are depicted underneath. Click here for file

Additional file 4 US abundance in the coding fraction and total DNA of 5 representative Pasteurellaceae genomes. Counts of USs and rcUSs in the genomes of five Pasteurellaceae species (with their accession number and coding density according to NCBI), the counts in the coding sequences (CDS), the total genomic count (Total count), the percentage of motifs found in the CDS (% of total) and the counts of motifs found by Redfield et al. (2006). Click here for file
