# Supplementary material for: An intragenic distribution bias of DNA uptake sequences in Pasteurellaceae and Neisseriae
Source: Biol Direct. 2008 Mar 27;3:12. doi: 10.1186/1745-6150-3-12 (PMC2346458; doi:10.1186/1745-6150-3-12)
Supplement: Additional file 1 — Total counts and fractions of the US in the genes of 7 Pasteurellaceae genomes. Total counts and percentages of the different motifs (the US 5'-AAAGTGCGGT-3' and rcUS 5'-ACCGCACTTT-3') in the coding regions of 7 Pasteurellaceae genomes (the accession numbers are given for each strain). [file 1745-6150-3-12-S1.doc]

| **Quintile (%)** | ***H. influenzae* Rd KW20 (NC000907)** | | ***P. multocida subsp. multocida* str. Pm70 (NC002663)** | | ***H. somnus* 129PT**  **(NC008309)** | | ***M. succiniciproducens* MBEL55E (NC006300)** | | ***H. influenzae* PittGG (NC009567)** | | ***H influenzae* 86-028NP (NC007146)** | | ***H. influenzae* PittEE (NC009566)** | | ***A. succinogenes* 130Z (NC009655)** | |
| --- | --- | --- | --- | --- | --- | --- | --- | --- | --- | --- | --- | --- | --- | --- | --- | --- |
|  | **US** | **rcUS** | **US** | **rcUS** | **US** | **rcUS** | **US** | **rcUS** | **US** | **rcUS** | **US** | **rcUS** | **US** | **rcUS** | **US** | **rcUS** |
| **1 (0-20)** | **48** | **122** | **38** | **72** | **44** | **88** | **63** | **125** | **44** | **112** | **53** | **129** | **44** | **108** | **38** | **130** |
| **2 (20-40)** | **48** | **57** | **23** | **27** | **56** | **48** | **61** | **62** | **48** | **53** | **49** | **63** | **44** | **60** | **54** | **49** |
| **3 (40-60)** | **65** | **87** | **34** | **42** | **69** | **60** | **70** | **79** | **46** | **73** | **63** | **96** | **56** | **82** | **53** | **72** |
| **4 (60-80)** | **48** | **59** | **33** | **29** | **42** | **47** | **60** | **60** | **48** | **57** | **55** | **64** | **48** | **55** | **62** | **43** |
| **5 (80-100)** | **77** | **50** | **45** | **42** | **67** | **39** | **59** | **70** | **63** | **45** | **67** | **49** | **59** | **48** | **70** | **51** |
| **total** | **286** | **375** | **173** | **212** | **278** | **282** | **313** | **396** | **249** | **340** | **287** | **401** | **251** | **353** | **277** | **345** |
| **p-value*** | **0.015** | **0** | ***0.11*** | **4.5E-06** | **0.023** | **3.0E-5** | ***0.87*** | **1.8E-7** | ***0.33*** | **0** | ***0.43*** | **0** | ***0.436*** | **7.7E-07** | **0.036** | **0** |
|  |  |  |  |  |  |  |  |  |  |  |  |  |  |  |  |  |
| **1 (0-20)** | **16.78** | **32.53** | **21.97** | **33.96** | **15.83** | **31.21** | **20.38** | **31.39** | **17.67** | **32.94** | **18.47** | **32.17** | **17.53** | **30.59** | **13.71** | **37.68** |
| **2 (20-40)** | **16.78** | **15.20** | **13.29** | **12.74** | **20.14** | **17.02** | **19.43** | **15.70** | **19.28** | **15.59** | **17.07** | **15.71** | **17.53** | **17.00** | **19.49** | **14.20** |
| **3 (40-60)** | **22.73** | **23.20** | **19.65** | **19.81** | **24.82** | **21.28** | **22.29** | **20.00** | **18.47** | **21.47** | **21.95** | **23.94** | **22.31** | **23.23** | **19.13** | **20.87** |
| **4 (60-80)** | **16.78** | **15.73** | **19.08** | **13.68** | **15.11** | **16.67** | **19.11** | **15.19** | **19.28** | **16.76** | **19.16** | **15.96** | **19.12** | **15.58** | **22.38** | **12.46** |
| **5 (80-100)** | **26.92** | **13.33** | **26.01** | **19.81** | **24.10** | **13.83** | **18.79** | **17.72** | **25.30** | **13.24** | **23.34** | **12.22** | **23.51** | **13.60** | **25.27** | **14.78** |
| **total** | **100%** | **100%** | **100%** | **100%** | **100%** | **100%** | **100%** | **100%** | **100%** | **100%** | **100%** | **100%** | **100%** | **100%** | **100%** | **100%** |

Additional file 1. Total counts and percentages of the different motifs (the US 5’-AAAGTGCGGT-3’ and rcUS 5’-ACCGCACTTT-3’) in the coding regions of 7 Pasteurellaceae genomes (the accession numbers are given for each strain).

*) The p-values represent the chance of rejection of the null-hypothesis, i.e., that the sequence motifs are distributed proportionally over all five quintiles. The p-values are based on chi-square tests with 4 degrees of freedom, for each column independently, to test whether each motif was equally distributed amongst all gene quintiles in each genome. P-values >0.05 are italicized.
