# Supplementary figures and images for: An intragenic distribution bias of DNA uptake sequences in Pasteurellaceae and Neisseriae
Source: Biol Direct. 2008 Mar 27;3:12. doi: 10.1186/1745-6150-3-12 (PMC2346458; doi:10.1186/1745-6150-3-12)

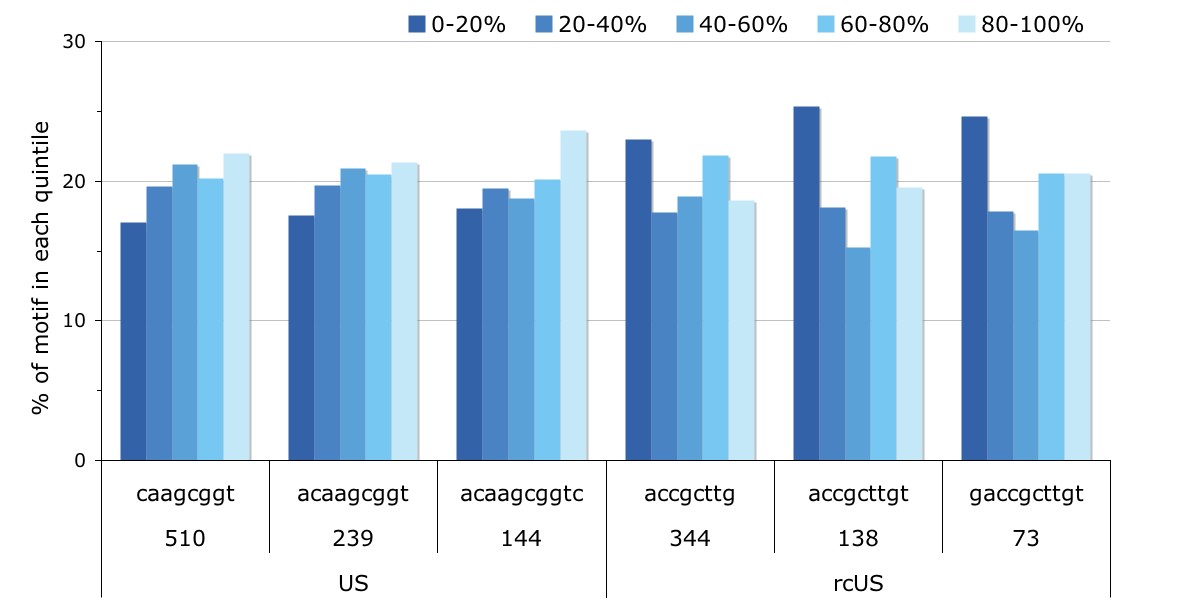

Supplement: Additional file 2 — The intragenic distribution of the Actinobacillus pleuropneumoniae uptake sequence. The intragenic distribution of the alternative US (left three sub-graphs, increasing the motif stringency from 8 to 10 bases) and its reverse complement (next three sub-graphs, increasing the motif stringency from 8 to 10 bases) in the protein coding regions of Actinobacillus pleuropneumoniae (accession number NC_009053). The counts of the motifs are depicted underneath. [file 1745-6150-3-12-S2.jpeg]

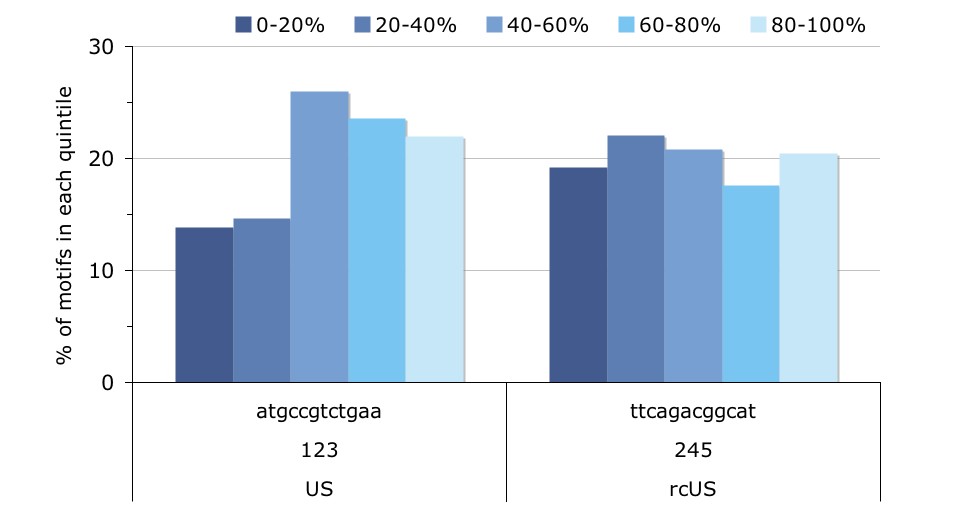

Supplement: Additional file 3 — The intragenic distribution of the Neisseria meningitidis uptake sequence. The intragenic distribution of the US and its reverse complement (rcUS) in the protein coding regions of Neisseria meningitidis MC58 (accession number NC_003112). Genes that start with the US are excluded. The sequence and the counts of the motifs are depicted underneath. [file 1745-6150-3-12-S3.jpeg]
