# Supplementary material for: An intragenic distribution bias of DNA uptake sequences in Pasteurellaceae and Neisseriae
Source: Biol Direct. 2008 Mar 27;3:12. doi: 10.1186/1745-6150-3-12 (PMC2346458; doi:10.1186/1745-6150-3-12)
Supplement: Additional file 4 — US abundance in the coding fraction and total DNA of 5 representative Pasteurellaceae genomes. Counts of USs and rcUSs in the genomes of five Pasteurellaceae species (with their accession number and coding density according to NCBI), the counts in the coding sequences (CDS), the total genomic count (Total count), the percentage of motifs found in the CDS (% of total) and the counts of motifs found by Redfield et al. (2006). [file 1745-6150-3-12-S4.doc]

Additional file 4. Counts of (rc)USs in the genomes of five Pasteurellaceae species (with their accession number and coding density according to NCBI), the counts in the coding sequences (CDS), the total genomic count (Total count), the percentage of motifs found in the CDS (% of total) and the counts of motifs found by Redfield et al. (2006).

*) Small discrepancies between these motif counts and those of Redfield et al. could be due to genome updates in NCBI

| **Strain** | **H. influenzae Rd KW20** | | **P. multocida subsp. multocida str. Pm70** | | **H. somnus 129PT** | | **M. succiniciproducens MBEL55E** | | **A. succinogenes 130Z** | |
| --- | --- | --- | --- | --- | --- | --- | --- | --- | --- | --- |
| **Acession number** | **NC_000907** | | **NC_002663** | | **NC_008309** | | **NC_006300** | | **NC_009655** | |
| **Coding density** | **84%** | | **88%** | | **88%** | | **89%** | | **87%** | |
|  |  |  |  |  |  |  |  |  |  |  |
|  | **CDS** | **Genome** | **CDS** | **Genome** | **CDS** | **Genome** | **CDS** | **Genome** | **CDS** | **Genome** |
| US | 286 | 565 | 173 | 345 | 278 | 391 | 313 | 637 | 277 | 658 |
| rcUS | 375 | 550 | 212 | 355 | 282 | 404 | 396 | 660 | 345 | 670 |
| Total count | 661 | 1115 | 385 | 700 | 560 | 795 | 709 | 1297 | 622 | 1328 |
| % of total | 59% |  | 55% |  | 70% |  | 55% |  | 47% |  |
| Redfield et al.  (2006) |  | 1115 |  | 700 |  | 776* |  | 1297 |  | NA |
